# Effect of COVID-19 vaccination on the outcome of *in vitro* fertilization: A systematic review and meta-analysis

**DOI:** 10.3389/fpubh.2023.1151999

**Published:** 2023-04-03

**Authors:** Linyu Zhang, Xinrui Sun, Ruohan Wang, Fang Ma

**Affiliations:** ^1^Key Laboratory of Birth Defects and Related Diseases of Women and Children, Center for Translational Medicine, Ministry of Education, West China Second University Hospital, Sichuan University, Chengdu, Sichuan, China; ^2^Department of Obstetrics and Gynecology, West China Second Hospital, Sichuan University, Chengdu, Sichuan, China

**Keywords:** *in vitro* fertilization, COVID-19, vaccine, pregnancy, meta-analysis, coronavirus disease 2019, severe acute respiratory syndrome coronavirus 2 infection

## Abstract

**Background:**

Universal COVID-19 vaccination programs are now recommended in several countries and represent the most effective preventive measure against COVID-19. However, some reports suggest that vaccination may cause infertility or have adverse effects on pregnancy. Conflicting reports have led to vaccine hesitancy in women planning pregnancy.

**Purpose:**

To determine whether vaccination against COVID-19 affects *in vitro* fertilization (IVF) outcomes, we conducted a meta-analysis.

**Method:**

A systematic search was conducted using PubMed, Embase, MEDLINE, and Web of Science databases for all published literature on COVID-19 vaccines and outcomes of IVF. International Prospective Register of Systematic Reviews registration was completed on September 13, 2022 (CRD42022359771).

**Results:**

We analyzed 20 studies totaling 18,877 individual cases undergoing IVF. COVID-19 vaccination had significant effect on clinical and ongoing pregnancy rate (risk ratio (RR): 0.97; 95% confidence interval (CI): 0.94–0.99; RR: 0.93; 95% CI: 0.87–0.99). These outcomes did not differ between vaccinated and unvaccinated individuals: biochemical pregnancy rate (RR: 0.95; 95% CI: 0.88–1.03), implantation rate (RR: 1.02; 95%CI: 0.97–1.07; *P* = 0.41), the number of oocytes (mean difference (MD): 0.12; 95% CI: −0.65–0.88) and MII/mature oocytes recovered (MD: 0.27; 95% CI: −0.36–0.90), blastocysts rate (MD: 0.01; 95% CI: −0.04, 0.06), and fertilization rate (MD: 1.08; 95% CI: −0.57, 2.73).

**Conclusion:**

Our findings suggest that vaccination against COVID-19 does not adversely affect the biochemical pregnancy rates; number of oocytes and MII/mature oocytes obtained; implantation, blastocysts; and fertilization rates in women undergoing IVF treatment. Subgroup analysis showed that the mRNA vaccine had no statistical significance on all indexes (clinical, biochemical, or ongoing pregnancy rates; implantation, blastocysts, or fertilization rates; and the number of oocytes and MII/mature oocytes). The findings of this meta-analysis are anticipated to increase the willingness of women planning IVF treatment to receive COVID-19 vaccination and provide evidence-based medical guidance for the development and implementation of guidelines.

**Systematic review registration:**

https://www.crd.york.ac.uk/PROSPERO/, identifier: CRD42022359771.

## 1. Introduction

The coronavirus disease 2019 (COVID-19) pandemic, caused by severe acute respiratory syndrome coronavirus 2 (SARS-CoV-2), is an infectious disease that continues to threaten human life and health. Globally, more than 6.5 million COVID-19-related deaths have been reported to the WHO, according to the uploaded Big Data count (1). Currently, there are no specific antiviral drugs to treat COVID-19, thus, vaccines against COVID-19 are the most promising preventive measure (2). As of February 22, 2023, more than 13.2 billion doses of COVID-19 vaccines had been administered worldwide (1). High rates of COVID-19 vaccination and thus, herd immunity, will be key to containing the pandemic (3); however, some reports suggest that vaccination may cause infertility or have adverse effects on pregnancy (4–6). Abbas-Hanif et al. (7) recommended that the safety of COVID-19 vaccines be evaluated during pregnancy, raising concerns for pregnant women and those planning *in vitro* fertilization (IVF) treatment (7). A meta-analysis of pregnant women revealed that only 47% of women intended to receive the COVID-19 vaccine during pregnancy, and women planning IVF treatment were also hesitant to receive the COVID-19 vaccine (8). Another meta-analysis reported that COVID-19 vaccination during pregnancy did not increase the risk of adverse perinatal outcomes but reduced the risk of stillbirth (9). A large retrospective cohort study found that COVID-19 vaccination during pregnancy was not significantly associated with an increased risk of adverse pregnancy outcomes compared with no vaccination during pregnancy (10). Moreover, despite the large number of studies investigating the effects of COVID-19 vaccination on IVF outcomes, no systematic review or meta-analysis of the observed results has been conducted.

We conducted a systematic review and meta-analysis of published observational studies to explore the impact of COVID-19 vaccination on IVF outcomes and to identify differences in clinical, biochemical, and ongoing pregnancy rates; implantation, blastocyst, and fertilization rate; and the number of oocytes and MII/mature oocytes recovered between vaccinated and unvaccinated individuals.

## 2. Materials and methods

This study was performed in accordance with the Preferred Reporting Items for Systematic Reviews and Meta-Analyses (PRISMA) Statement (11). The protocol has been registered on the PROSPERO platform (registration no. CRD42022359771).

### 2.1. Literature search strategy

PubMed, Embase, MEDLINE, and Web of Science databases were searched for literature published between January 1, 2020 and February 24, 2023, using a combination of the following search queries: COVID-19 vaccine AND (*in vitro* fertilization OR IVF), without language restrictions. Import all published articles retrieved from these databases into the EndNote software X9.3.3 and then use this software to remove duplicates articles. Two investigators (LZ and XS) independently read the article titles and abstracts according to the inclusion and exclusion criteria set in advance and conducted a full text analysis of the articles that met the criteria. Additionally, the reference lists of the relevant articles were manually searched.

### 2.2. Eligibility criteria

- Participants: Population vaccinated against COVID-19 undergoing IVF.
- Exposure: Women who have been vaccinated against COVID-19 and are not infected with COVID-19.
- Comparison: Women who have not been vaccinated against COVID-19 and are not infected with COVID-19.
- Outcomes: Clinical, biochemical, or ongoing pregnancy rates; implantation, blastocysts, or fertilization rate; and number of oocytes and MII/mature oocytes.
- Study types: All cohort or case-control studies. Journal articles, conference abstracts, and letters that described relevant methods and results were included. Animal studies, reviews, case reports, and editorials were excluded.

We excluded studies that included people infected with COVID-19. For studies that clearly delineate infected, uninfected, vaccinated, and unvaccinated, we included only vaccinated and unvaccinated data; In addition, we chose to include relevant data for the study that divided only those vaccinated and those not vaccinated.

### 2.3. Data extraction

The evaluation was not influenced by the authorized institution or journal related to the study. Data were independently extracted by two researchers (LYZ and XRS), and disagreements were settled by another author (FM). The extracted information included basic study information, vaccine type, transplantation method, and outcomes. Original article authors were contacted if the article data was unintelligible.

For the preliminary analysis we included data on clinical, biochemical, or ongoing pregnancy rates; implantation, blastocysts, or fertilization rates; and the number of oocytes and MII/mature oocytes for IVF in all women vaccinated against COVID-19. These outcomes are defined as follows:

*Clinical pregnancy*: The presence of an intrauterine gestational sac observed by ultrasound scanning and detection of serum human chorionic gonadotropin.

*Biochemical pregnancy*: Pregnancy with elevated human chorionic gonadotropin levels in the absence of an intrauterine gestational sac.

*Ongoing pregnancy*: Pregnancy that lasts for more than 12 weeks with a viable fetus

*Implantation rate*: Number of gestational sacs observed divided by the number of embryos transferred.

*Blastocyst*: Preimplantation stage of embryonic development, which occurs about 5–6 days after fertilization.

*Fertilization*: A series of biological processes that begin with the identification of a sperm with a mature oocyte and lead to the formation of a prokaryote (12).

*Oocytes*: The female gamete.

*Mature oocytes*: Oocytes in the metaphase of meiosis, displaying the first polar body and having the ability to combine with sperm.

### 2.4. Quality assessment

Quality assessment was independently performed by LYZ and RHW. A meta-analysis of non-randomized studies using Newcastle–Ottawa scale (NOS) scores was conducted to evaluate the included cohort studies (13). The risk of study bias was assessed in terms of population selection, comparability between exposed and non-exposed groups, and reliability of outcomes.

### 2.5. Statistical analysis

Data analysis was performed using Cochrane Review Manager 5.3 (The Nordic Cochrane Center, The Cochrane Collaboration 2014; Copenhagen, Denmark) (14). Considering the different types of studies included (prospective and retrospective cohort studies), we chose the random-effects model (15). The Mantel–Haenszel method was used for meta-analysis of dichotomous variable data (clinical, biochemical, and ongoing pregnancy rates and implantation rate) and the inverse-variance method was used to merge continuous variable data (number of oocytes, number of MII/mature oocytes, blastocysts rate, and fertilization rate). The Q test and *I*^2^ index values were evaluated using heterogeneity. The effect of the COVID-19 vaccination on pregnancy outcomes after IVF was expressed as a risk ratio (RR), and the prediction range of the RR was expressed as a 95% confidence interval (CI). Mean Difference (MD) and 95% CI were used to show the effect and prediction range of the COVID-19 vaccine on the number of oocytes and MII/mature oocytes, blastocysts rate, and fertilization rate. *P* ≤ 0.05 was considered statistically significant. Subgroup analyses were performed for the main types of vaccines administered – mRNA, inactivated virus, or viral vector. Furthermore, to evaluate the robustness of the effect size, we performed sensitivity analyses by excluding one study so that the impact of each study on the pooled effect size could be assessed. Funnel plots were used to analyze publication bias in the outcomes of more than ten studies (16). Publication bias was assessed for indicators using Egger's test in Stata 15.1 (Stata Corp., College Station, TX, USA). The trim and fill analysis was used to analyze the indicators with publication bias (17).

## 3. Results

### 3.1. Literature search

**Figure 1:**
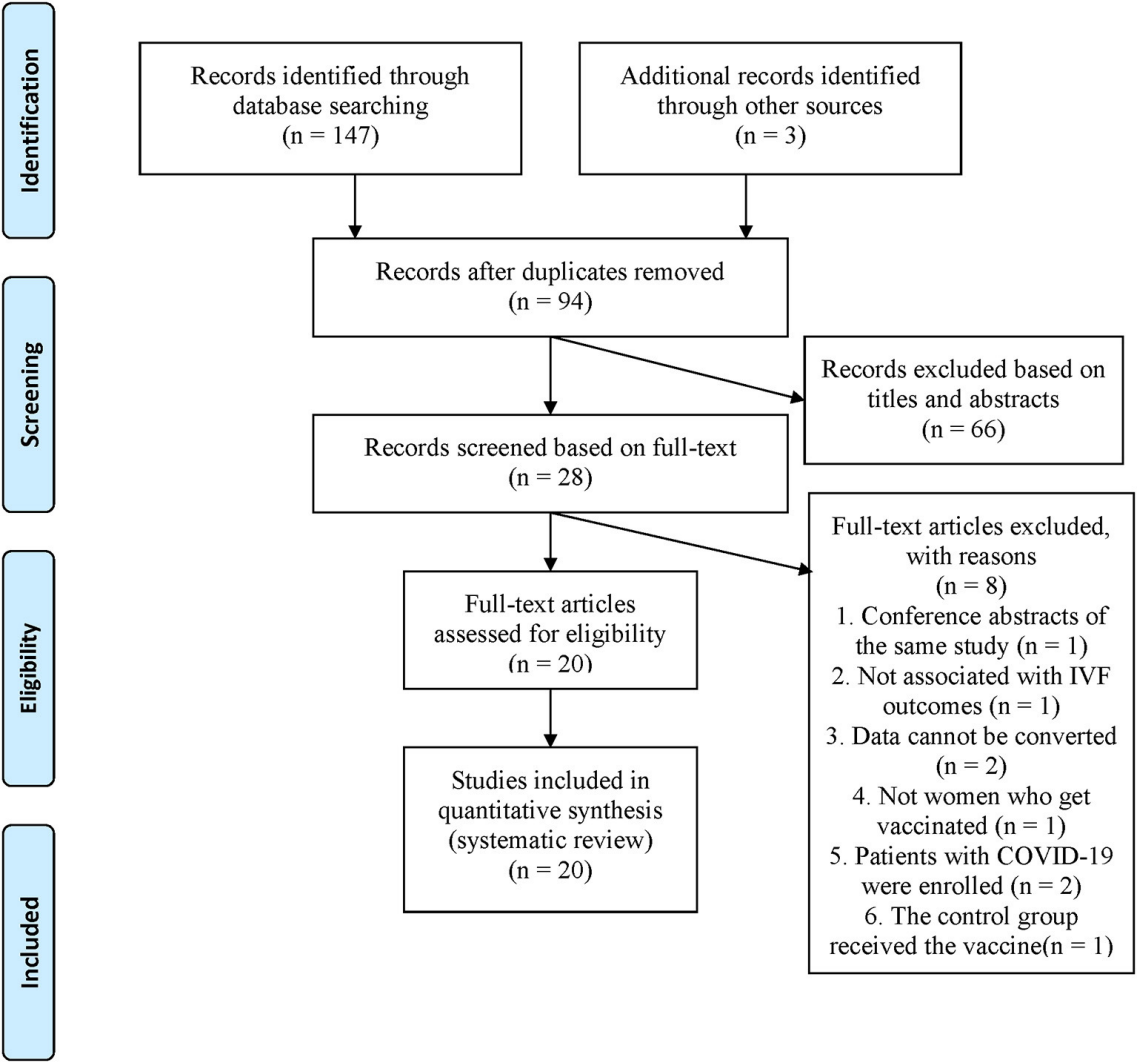
PRISMA flow diagram.

PubMed, Embase, MEDLINE, and Web of Science, were searched, and 147 articles were retrieved. Three studies were manually searched by screening the references included in the full text or related reviews. After elimination of duplicate literature, 94 articles remained. LYZ and XRS independently read the article titles and abstracts, screening them according to the inclusion and exclusion criteria, resulting in 28 valid articles which were included for further analysis. The examiners analyzed the full text of the 28 articles and excluded eight articles that did not fully meet the requirements, ending with a total of 20 articles that were analyzed (Figure 1).

### 3.2. Patient characteristics

The final 20 studies included 18,877 women with median age range from 30.4 to 38.7 years undergoing IVF mainly from China, Israel, Spain, the United States, and Italy. Among them, one study compared the IVF outcomes before- and after vaccination (18). The women were sorted into a vaccinated or unvaccinated group based on their COVID-19 vaccination status. First author, year, country, study design, sample size, population, vaccine type, transfer strategy, and outcomes (clinical, biochemical, and ongoing pregnancy rates; implantation, blastocysts, and fertilization rates; and the number of oocytes and MII/mature oocytes) are summarized in Table 1.

**Table 1:** Characteristics of studies included this systematic review and meta-analysis.

**First author, year**	**Country**	**Study design**	**Sample size**	**Median age (years)**	**Population**	**Vaccine type**	**Transfer strategy**	**Outcomes**
Bentov et al. (19)	Israel	Prospective cohort study	I: 9 C: 14	I: 35.3 C: 32.5	I: received vaccine C: unvaccinated	mRNA vaccine (BNT162b2)	NA	No. of oocytes No. of mature oocytes
Aharon et al. (20)	United States	Retrospective cohort study	I: 214 C: 733	I: 36.5 C: 36.5	I: received vaccine C: unvaccinated	mRNA vaccine (BNT162b2 or mRNA-1273)	Single euploid frozen-thawed embryo transfer	Clinical and ongoing pregnancy rates No. of MII/mature oocytes Blastocysts and fertilization rates
Aizer et al. (21)	Israel	Retrospective cohort study	I: 115 C: 93	I: 30.4 C: 30.7	I: received vaccine (between Jan and Aug 2021) C: unvaccinated (between Jan and Aug 2021)	mRNA vaccine (BNT162b2)	Frozen-thawed embryo transfer	Clinical and ongoing pregnancy rates Implantation rates
Avraham et al. (22)	Israel	Retrospective cohort study	I: 128 C: 133	I: 35.41 C: 30.7	I: received vaccine C: unvaccinated	mRNA vaccine (BNT162b2)	Fresh embryo transfer freeze-all cycles	Clinical and biochemical pregnancy rates No. of oocytes Fertilization rates
Brandão et al. (23)	Spain	Retrospective cohort study	I: 890 C: 3272	I: 38.7 C: 38.2	I: received 1-2 doses of vaccine C: underwent embryo transfer in the year before the pandemic	mRNA vaccine (BNT162b2 or mRNA-1273)	Fresh embryo transfers cryopreserved embryo transfers	Clinical pregnancy rates implantation rates
Castiglione Morelli et al. (18)	Italy	Prospective cohort study	I: 6 C: 9	I: 36.2 C: 36.2	I: received vaccine C: unvaccinated in the year before the pandemic	mRNA vaccine (BNT162b2 or mRNA-1273) Viral vector vaccine (Oxford/AstraZeneca vaccine)	Fresh embryo transfer	No. of oocytes No. of MII/mature oocytes
Dong et al. (24)	China	Prospective cohort study (PSM)	I: 155 C: 340	I: 32.9 C: 32.69	I: received two doses of vaccine C: unvaccinated	Inactivated SARS-CoV-2 vaccines	Fresh embryo transfer frozen embryo transfer	Clinical and biochemical pregnancy rates No. of oocytes Blastocysts and fertilization rates
Huang et al. (25)	China	Retrospective cohort study (PSM)	I: 146 C: 584	I: 33.6 C: 33.4	I: received two doses of vaccine C: unvaccinated	Inactivated SARS-CoV-2 vaccines (Sinopharm or Sinovac)	Fresh embryo transfer frozen embryo transfer	Clinical and biochemical pregnancy rates implantation rates No. of oocytes No. of MII/mature oocytes Blastocysts and fertilization rates
Huang et al. (26)	China	Retrospective cohort study	I: 20 C: 25	I: 36.1 C: 35.9	I: vaccinated with two doses of vaccines C: unvaccinated	Inactivated SARS-CoV-2 vaccines (Sinopharm or Sinovac)	Frozen embryo transfer	Clinical and biochemical pregnancy rates No. of oocytes No. of MII/mature oocytes Blastocysts and fertilization rates
Jacobs et al. (27)	United States	Retrospective cohort study	I: 142 C: 138	I: 34 C: 33	I: vaccinated with one/two doses of vaccines C: unvaccinated	mRNA vaccine (mRNA-1273 or BNT162b2); Viral vector vaccine (Ad26.COV2. S)	Fresh embryo transfer	Clinical and ongoing pregnancy rates No. of oocytes Blastocysts and fertilization rates
Karavani et al. (28)	Israel	Retrospective cohort study	I: 69 C: 103	I: 35.4 C: 35.4	I: vaccinated with two doses of vaccines C: unvaccinated	mRNA vaccine (BNT162b2 or mRNA-1273)	Fresh embryo transfer	No. of oocytes No. of MII/mature oocytes
Wang et al. (29)	China	Retrospective cohort study	I: 460 C: 1036	I: 33.58 C: 33.13	I: vaccinated with two doses of vaccines C: unvaccinated	Inactivated SARS-CoV-2 vaccines (Sinopharm or Sinovac)	Frozen embryo transfer	Clinical pregnancy rates
Wu et al. (30)	China	Retrospective cohort study (PSM)	I: 239 C: 928	I: 33.8 C: 33.4	I: received vaccines C: unvaccinated	Inactivated SARS-CoV-2 vaccines	Fresh embryo transfer	Clinical, biochemical, and ongoing pregnancy rates implantation rates
Bosch et al. (31)	Spain	Prospective cohort study	I&C: 32	NA	I: vaccinated with two doses of vaccines C: unvaccinated	mRNA vaccines	NA	Clinical pregnancy rates
Cao et al. (32)	China	Retrospective cohort	I: 502 C: 1589	I: 32.43 C: 32.70	I: received vaccines C: did not receive vaccine	Inactivated vaccines	Frozen-thawed embryo transfer	Clinical, biochemical, and ongoing pregnancy rates
Chen et al. (33)	China	Retrospective cohort	I: 223 C: 268	I: 33.32 C: 32.81	I: received vaccines C: unvaccinated	Inactivated or recombinant vaccines	Frozen embryo transfer	Clinical pregnancy rates implantation rates No. of oocytes No. of MII/mature oocytes
Shi et al. (34)	China	Prospective cohort study	I: 667 C: 2385	I: 32.0 C: 31.0	I: received vaccines C: unvaccinated	Inactivated vaccines	Fresh embryo transfer	Clinical, biochemical, and ongoing pregnancy rates
Alder Lazarovits et al. (35)	Israel	Prospective cohort study	I: 75 C: 9	I: 32.9 C: 34.3	I: vaccinated and boosted, or vaccinated without the booster dose C: unvaccinated	mRNA vaccines	Fresh and thawed embryo transfer	Clinical pregnancy rates
Huang et al. (36)	China	Retrospective cohort study	I&C: 265	I: 31 C: 30.9	I: received vaccines C: unvaccinated	Inactivated vaccines	Frozen-thawed embryo transfer	Clinical and biochemical pregnancy rates implantation rates
Zhao et al. (37)	China	Retrospective cohort study	I: 781 C: 1851	NA	I: received vaccines C: unvaccinated	Inactivated vaccines	Fresh embryo transfer frozen embryo transfer	Clinical pregnancy rates

C, control group; I, intervention group; MII, metaphase II; NA, not available; PSM, propensity score matching; SARS-CoV-2= severe acute respiratory syndrome coronavirus 2.

### 3.3. Quality assessment

NOS quality assessment scored more than or equal to 7 as high quality, 5–6 as medium quality, and < 5 as low quality (38). Overall, 19 of the 20 cohort studies (18–30, 32–37) were of high quality (NOS score ≥7). The remaining study (31) was of relatively poor quality, as summarized in Table 2. Some of the studies were unblinded (unable to know grouping during statistical results), and others had incomplete documentation of the results, hence, the reduced quality of these studies.

**Table 2:** Outcome of assessment of the quality of non-randomized studies using the Newcastle-Ottawa scale.

**Cohort studies**	**Selection**				**Comparability**		**Outcome**			
	**Representativeness of the exposed cohort**	**Selection of non-exposed cohort**	**Ascertainment of exposure**	**Outcome not presented at the start**	**Age and BMI**	**Most of** **additional** **factors**	**Assessment of outcome**	**Follow-up long enough**	**Adequacy of follow up**	**Total score**
Bentov et al. (19)	^*^	^*^	^*^	^*^	^*^	^*^	^*^	^*^	^*^	9/9
Aharon et al. (20)	^*^	^*^	^*^	^*^	^*^	^*^	^*^	^*^	^*^	9/9
Aizer et al. (21)	^*^	^*^	^*^	^*^	^*^	^*^	^*^	^*^	^*^	9/9
Avraham et al. (22)	^*^	^*^	^*^	^*^	^*^	^*^	-	^*^	^*^	8/9
Brandão et al. (23)	^*^	^*^	^*^	-	^*^	^*^	-	^*^	^*^	7/9
Castiglione Morelli et al. (18)	^*^	^*^	^*^	^*^	^*^	^*^	-	^*^	^*^	8/9
Dong et al. (24)	^*^	^*^	^*^	^*^	^*^	^*^	-	^*^	^*^	8/9
Huang et al. (25)	^*^	^*^	^*^	^*^	^*^	^*^	-	^*^	^*^	8/9
Huang et al. (26)	^*^	^*^	^*^	^*^	^*^	^*^	-	^*^	^*^	8/9
Jacobs et al. (27)	^*^	^*^	^*^	^*^	^*^	^*^	-	^*^	^*^	8/9
Karavani et al. (28)	^*^	^*^	^*^	^*^	^*^	^*^	^*^	^*^	^*^	9/9
Wang et al. (29)	^*^	^*^	^*^	^*^	^*^	^*^	-	^*^	^*^	8/9
Wu et al. (30)	^*^	^*^	^*^	^*^	^*^	^*^	^*^	^*^	^*^	9/9
Bosch et al. (31)	^*^	^*^	^*^	^*^	-	-	-	^*^	^*^	6/9
Cao et al. (32)	^*^	^*^	^*^	^*^	^*^	^*^	^*^	^*^	^*^	9/9
Chen et al. (33)	^*^	^*^	^*^	^*^	^*^	^*^	^*^	-	^*^	8/9
Shi et al. (34)	^*^	^*^	^*^	^*^	-	-	^*^	^*^	^*^	7/9
Alder Lazarovits et al. (35)	^*^	^*^	^*^	^*^	^*^	^*^	-	^*^	^*^	8/9
Huang et al. (36)	^*^	^*^	^*^	^*^	^*^	^*^	-	^*^	^*^	8/9
Zhao et al. (37)	^*^	^*^	^*^	^*^	^*^	^*^	-	^*^	^*^	8/9

A single asterisk (^*^) indicates 1 score, and dash (-) indicates 0 score.

### 3.4. Meta-analysis

**Figure 2:**
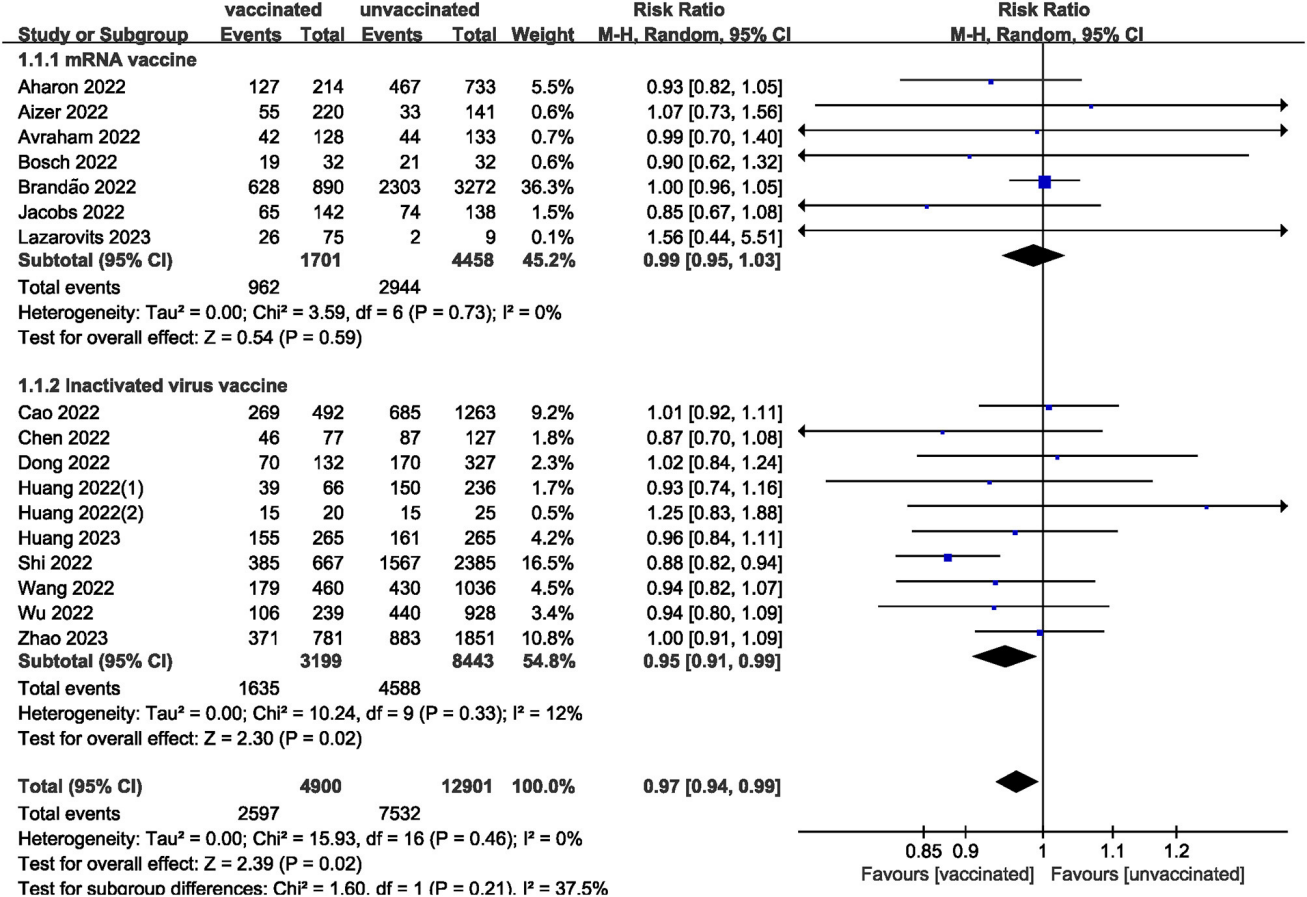
Forest plot of clinical pregnancy rate for vaccinated vs. unvaccinated.

**Figure 3:**
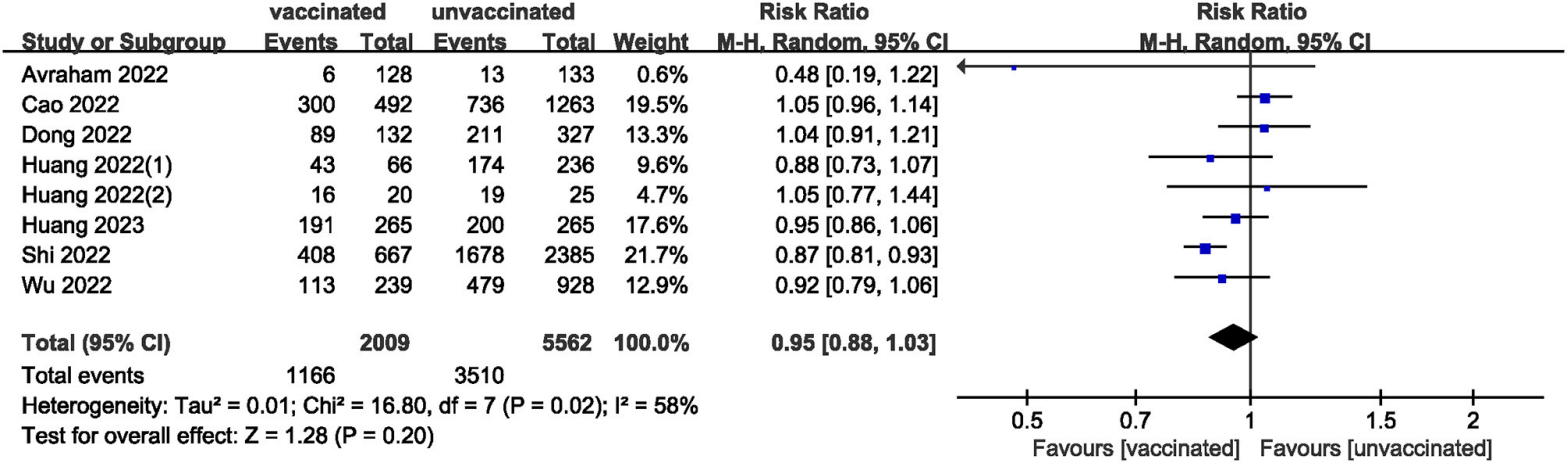
Forest plot of biochemical pregnancy rate for vaccinated vs. unvaccinated.

**Figure 4:**
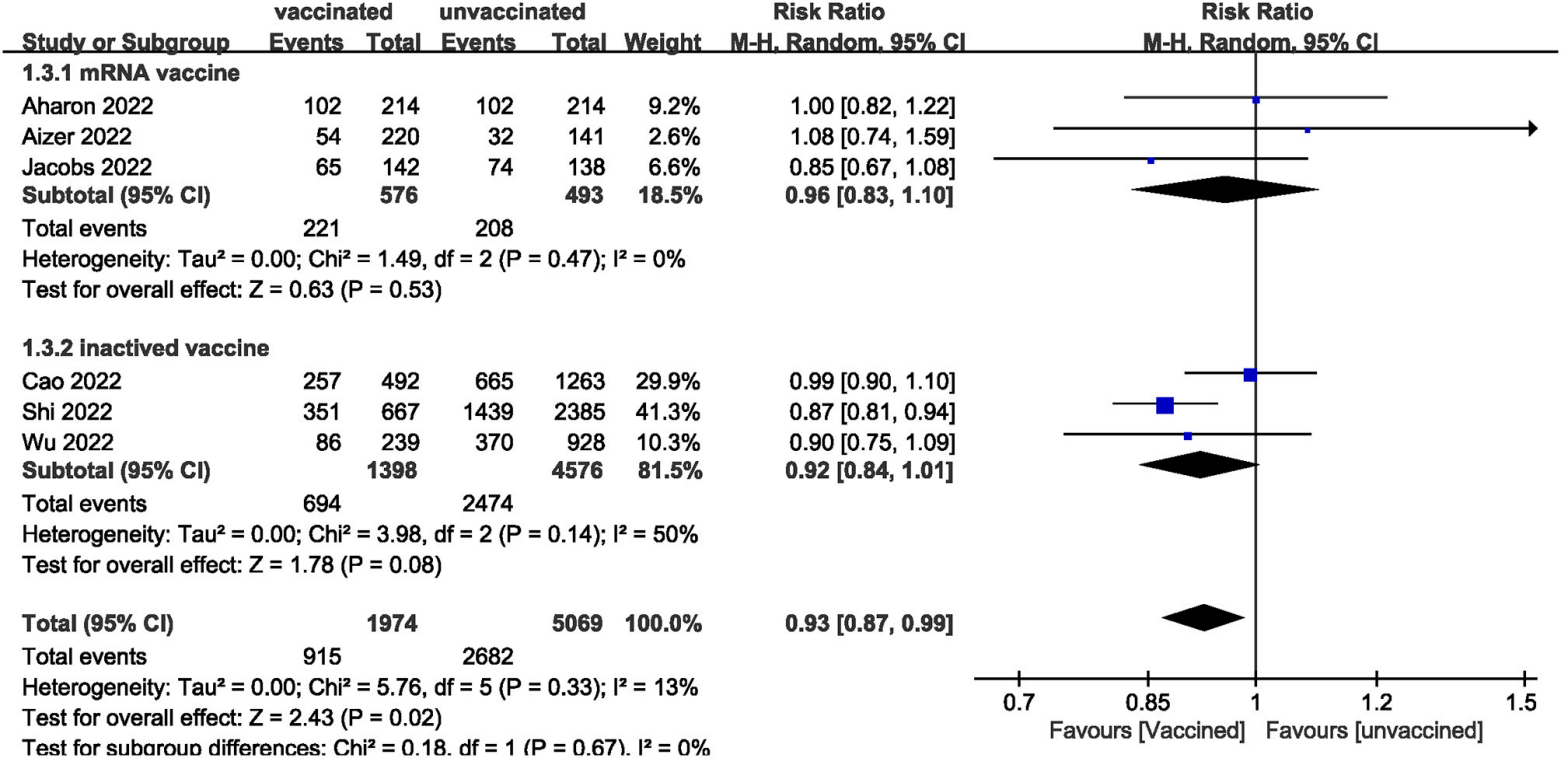
Forest plot of ongoing pregnancy rate for vaccinated vs. unvaccinated.

**Figure 5:**
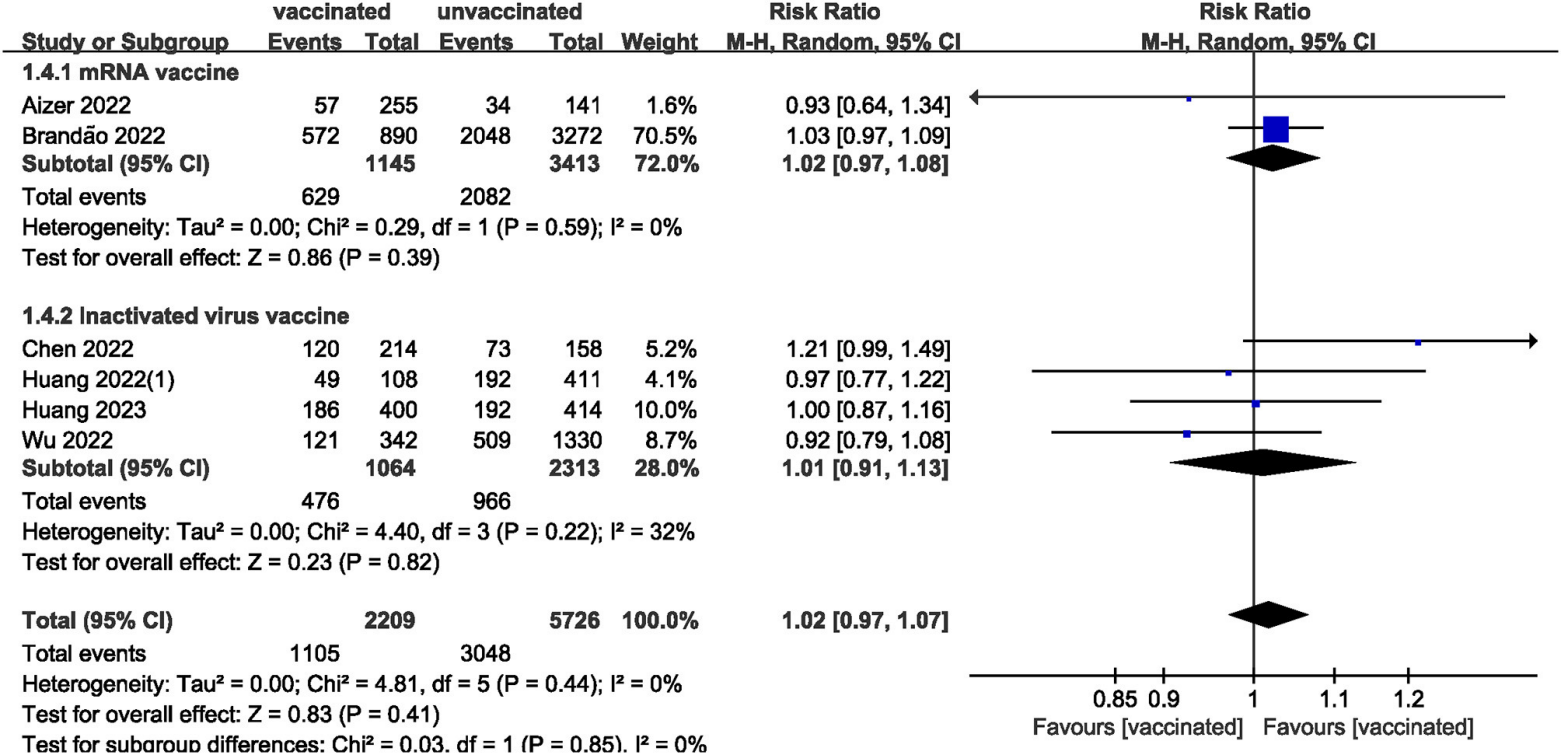
Forest plot of implantation rate for vaccinated vs. unvaccinated.

We pooled data from 17801 participants (Intervention group = 4,900; Control group = 12,901) from 17 studies (20–27, 29–37) compare clinical pregnancy outcomes between the vaccinated and unvaccinated groups and found statistical differences (RR: 0.97; 95% CI: 0.94–0.99; *P* = 0.02; Figure 2). Eight studies (22, 24–26, 30, 32, 34, 36) showed that the biochemical pregnancy rate was not affected by vaccination (RR: 0.95; 95% CI: 0.88–1.03; *P* = 0.20; Figure 3). Ongoing pregnancy rates were calculated from six studies (20, 21, 27, 30, 32, 34) and were found statistical differences in the vaccinated group than in the unvaccinated group (RR: 0.93; 95% CI: 0.87–0.99; *P* = 0.02; Figure 4). We compared the differences in implantation rate data from six studies (21, 23, 25, 30, 33, 36) between vaccinated and unvaccinated groups, and there were no differences (RR: 1.02; 95%CI: 0.97–1.07; *P* = 0.41; Figure 5). The Q test and *I*^2^ index showed minimal heterogeneity in pregnancy outcomes (clinical pregnancy rate *P* = 0.46, *I*^2^ = 0%; ongoing pregnancy rate *P* = 0.33, *I*^2^ = 13%; and implantation rate *P* = 0.44, *I*^2^ = 0%). Biochemical pregnancy rate has moderate heterogeneity (*P* = 0.02, *I*^2^ = 58%). Among the pooled indicators, the quality of the studies involved was at a high level (20–27, 29, 30, 32–37), except for the clinical pregnancy rate, which included a study with an NOS score of >7 (31), and are summarized in Table 3.

**Table 3:** Newcastle-Ottawa scale of each outcome.

**Outcome**	**Effect (95%CI)**	***I* ^2^**	**Newcastle-Ottawa scale of each study**
Clinical pregnancy rates	RR 0.97 (0.94, 0.99)	0	9,9,8,6,7,8,8,9,8,8,8,8,7,8,9,8,8
Biochemical pregnancy rates	RR 0.95 (0.88, 1.03)	58	8,9,8,8,8,8,7,9
Ongoing pregnancy rates	RR 0.93 (0.87, 0.99)	13	9,9,8,9,7,9
Implantation rates	RR 1.02 (0.97, 1.07)	0	9,7,8,8,8,9
No. of oocytes	MD 0.12 (−0.65, 0.88)	51	8,9,8,8,9,8,8,8,8
No. of MII/mature oocytes	MD 0.27 (−0.36, 0.90)	13	9,9,8,9,8,8,8
Blastocysts rates	MD 0.01 (−0.04, 0.06)	0	9,8,8,8,8
Fertilization rates	MD 1.08 (−0.57, 2.73)	36	9,8,8,8,8,8

CI, confidence interval; MD, Mean Difference; RR, risk ratio.

**Figure 6:**
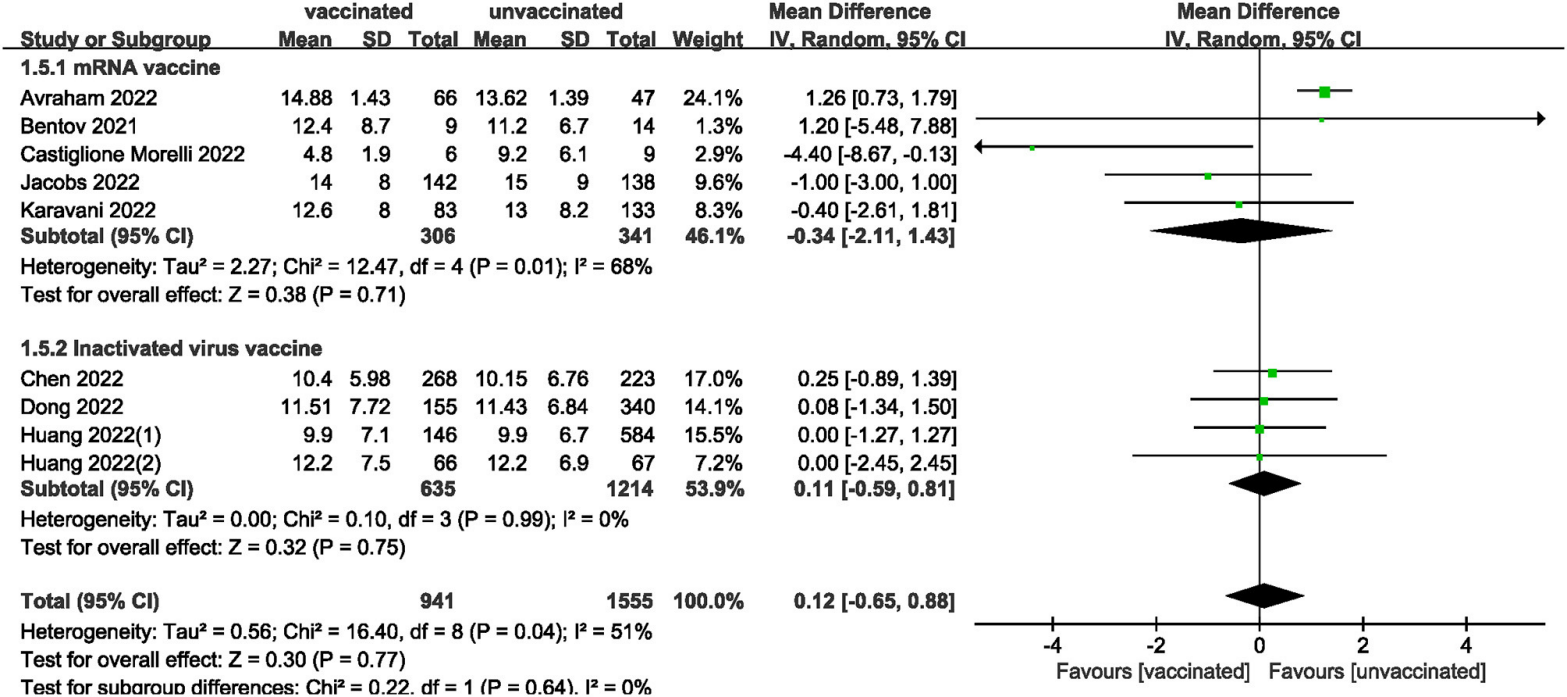
Forest plot of number of oocytes for vaccinated vs. unvaccinated.

**Figure 7:**
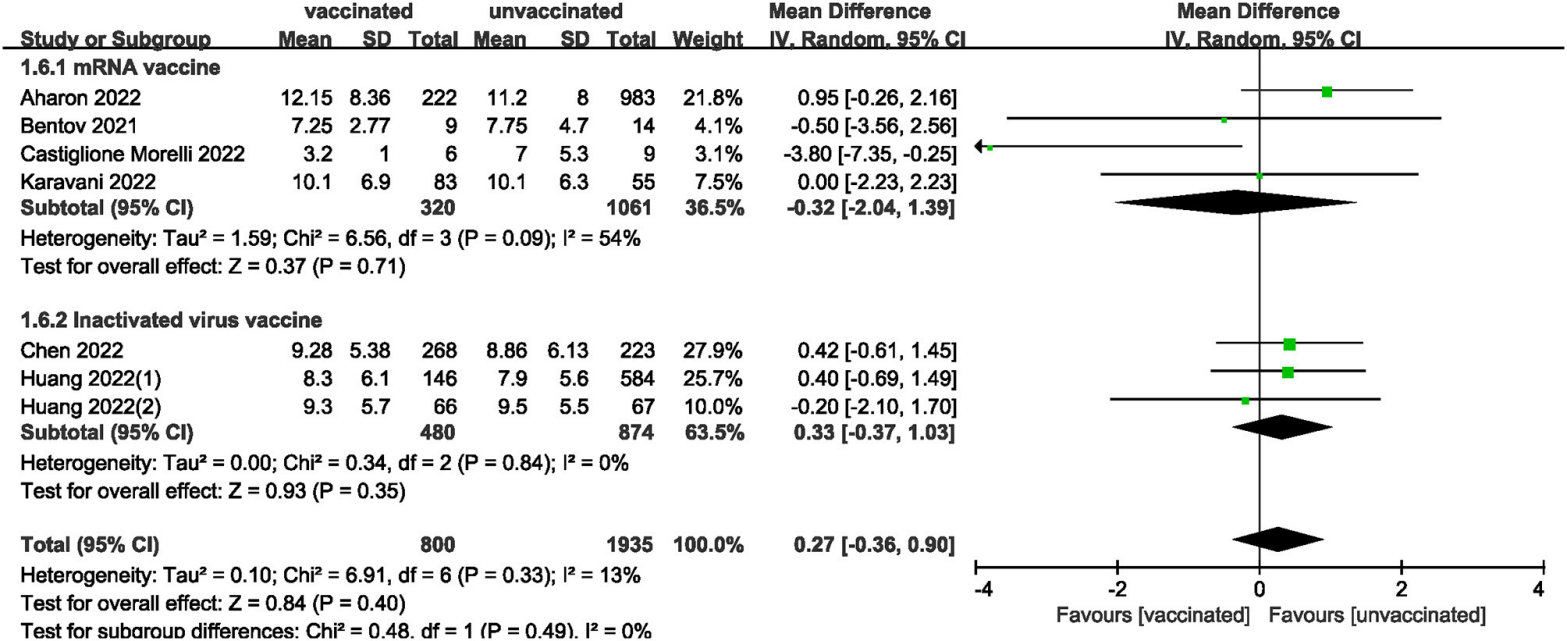
Forest plot of number of MII/mature oocytes for vaccinated vs. unvaccinated.

**Figure 8:**
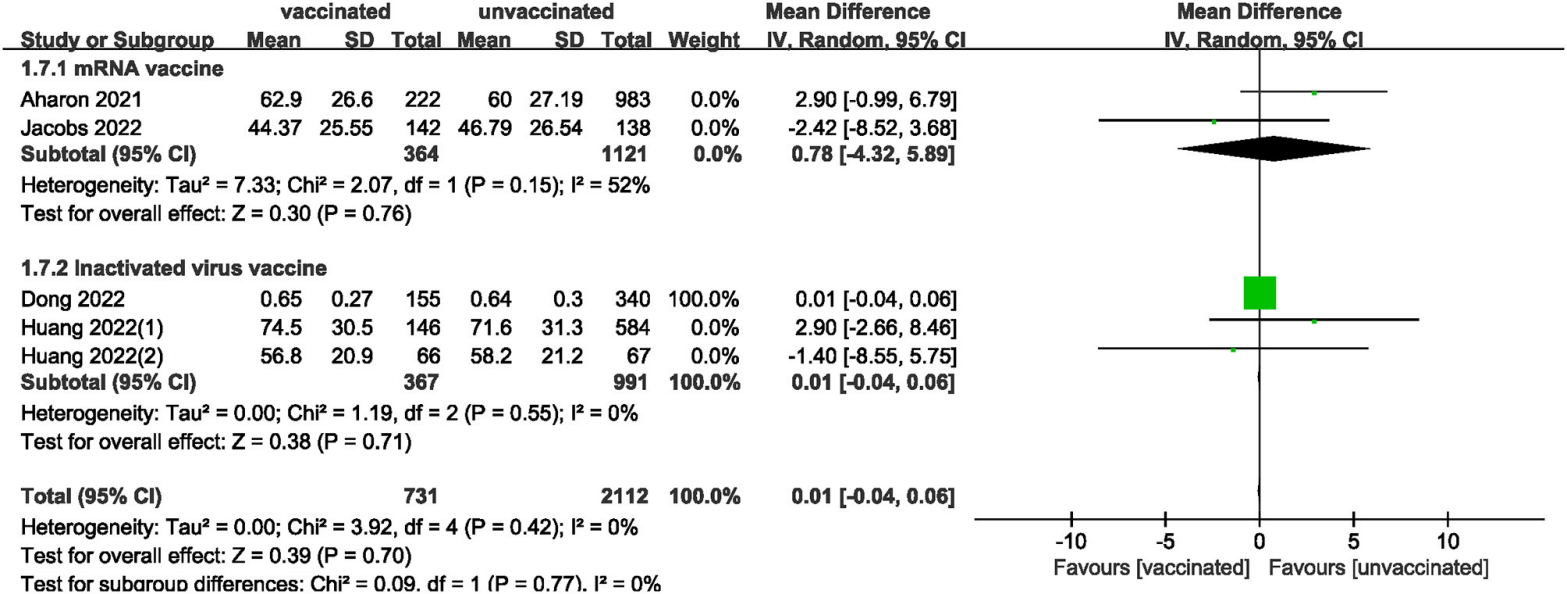
Forest plot of blastocysts rate for vaccinated vs. unvaccinated.

**Figure 9:**
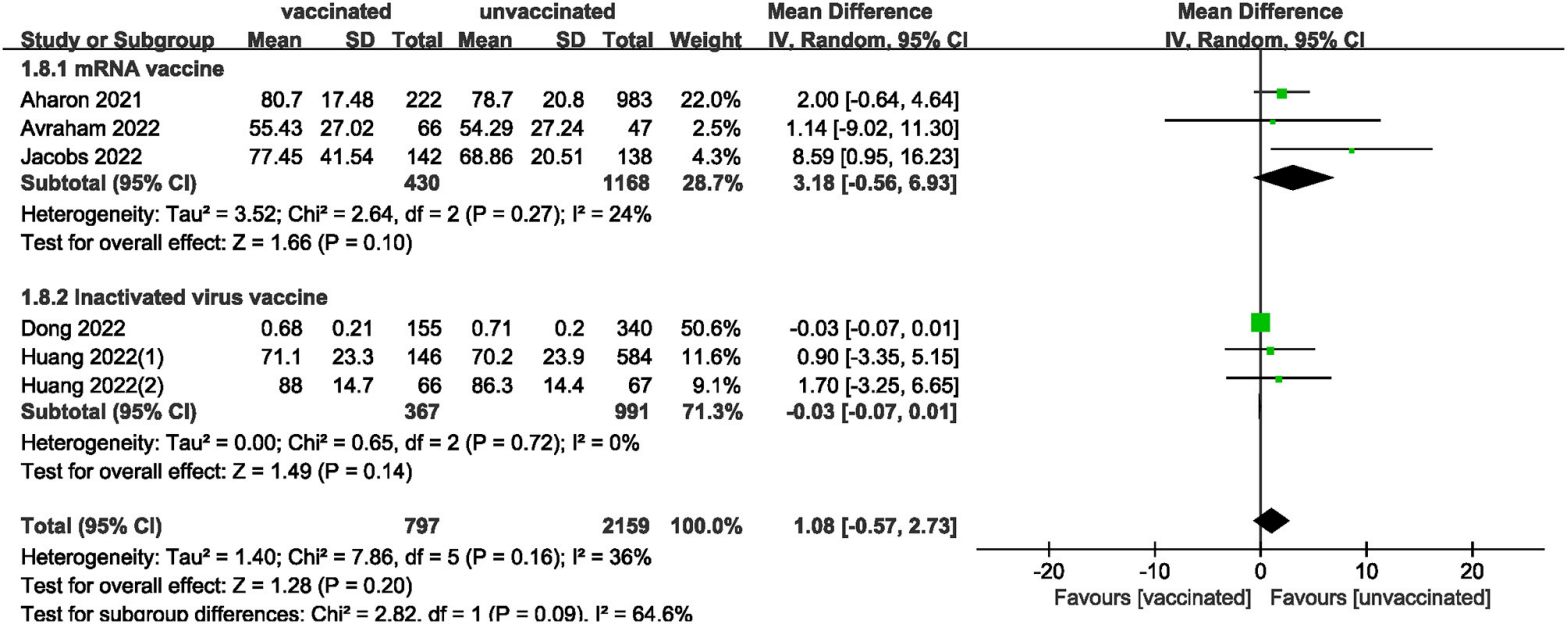
Forest plot of fertilization rate. for vaccinated vs. unvaccinated.

We also analyzed data on whether COVID-19 vaccines affected the number of oocytes and MII/mature oocytes, blastocysts rate, and fertilization rate. Data on the number of oocytes from nine studies (18, 19, 22, 24–28, 33) were combined, and the difference was not statistically significant (MD: 0.12; 95% CI: −0.65–0.88; *P* = 0.77; Figure 6). Moreover, there was no statistically significant difference in the number of MII/mature oocytes between the vaccinated and unvaccinated groups from seven studies (MD: 0.27; 95% CI: −0.36–0.90; *P* = 0.40; Figure 7) (18–20, 25, 26, 28, 33). The rates of blastocyst formation (20, 24–27, 33) and fertilization (20, 22, 24–27) were also not significantly different between the vaccinated and unvaccinated groups (MD: 0.01 vs. 1.08; 95% CI: −0.04–0.06 vs. −0.57–2.73; *P* = 0.70 vs. *P* = 0.20, respectively; Figures 8, 9). The Q test and *I*^2^ index of the number of oocytes (*P* = 0.04, *I*^2^ =51%) and MII/mature oocytes (*P* = 0.33, *I*^2^ =13%), blastocyst rate (*P* = 0.42, *I*^2^ =0%) and fertilization rate (*P* = 0.16, *I*^2^ =36%) showed low to moderate heterogeneity. The quality of the studies involved in the combined index is at a high level (18–20, 22, 24–28, 33), as summarized in Table 3.

The included studies consisted of women vaccinated with either an mRNA or inactivated virus vaccine. We performed additional analyses by subdividing the women based on the type of vaccine received. The analysis found no significant differences in all measures (clinical, biochemical, or ongoing pregnancy rates; implantation, blastocysts, or fertilization rates; and the number of oocytes and MII/mature oocytes) between the mRNA vaccinated and unvaccinated groups. However, a statistically significant difference was observed in clinical pregnancy rates between the inactivated virus vaccinated and unvaccinated groups.

### 3.5. Sensitivity analysis

The results showed that excluding any single study had no significant effect on the total effect size of number of oocytes and MII/mature oocytes; blastocyst formation, implantation, and fertilization rates. The total effect size for the clinical pregnancy rate (RR: 0.97; 95% CI: 0.94–0.99) changed when the study by Shi et al. (34) (RR: 0.98; 95% CI: 0.95–1.01) was excluded. Sensitivity analysis of biochemical pregnancy rate (RR: 0.95; 95% CI: 0.88–1.03) revealed that excluding Cao et al. (32) study from the meta-analysis changed the total effect size (RR: 0.93; 95% CI: 0.87–0.99). Excluding the studies by Jacobs et al. (27), Shi et al. (34), and Wu et al. (30) the total effect size for ongoing pregnancy rate (RR: 0.93; 95% CI: 0.87–0.99) changed (RR: 0.93 vs. 0.97 vs. 0.93; 95% CI: 0.87–1.00 vs. 0.90–1.04 vs. 0.86–1.01, respectively).

### 3.6. Publication bias

**Figure 10:**
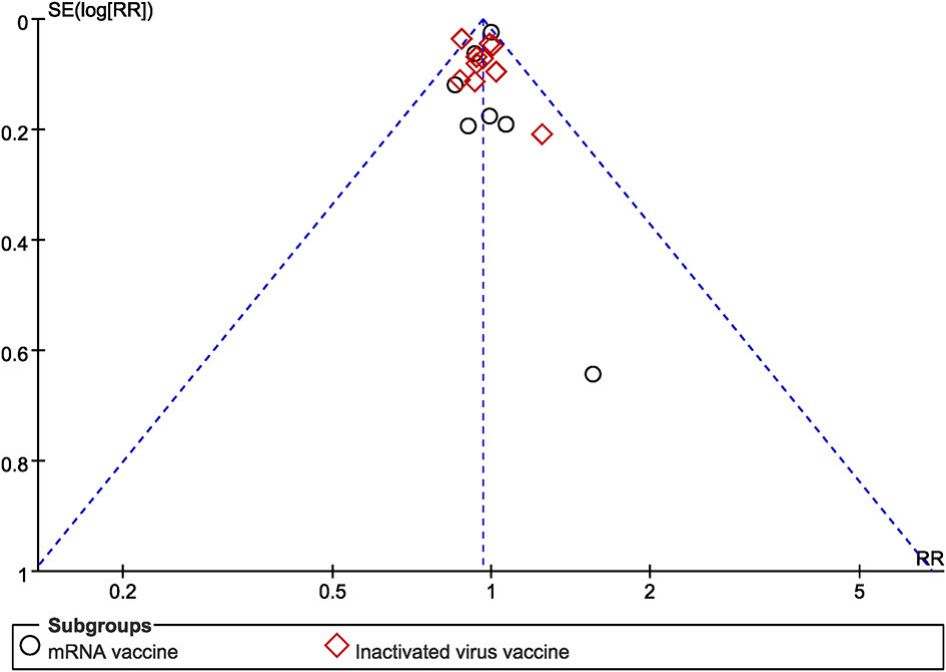
Funnel plot of clinical pregnancy rate.

The funnel plot of the studies included in the clinical pregnancy rate was roughly symmetric, with an Egger value of 0.968 (Figure 10). There was no publication bias in ongoing pregnancy rate, biochemical pregnancy rate, blastocysts rate, implantation rate and fertilization rate, with Egger values of 0.718, 0.886, 0.589, 0.844 and 0.053, respectively. However, there was publication bias in the number of oocytes and MII/mature oocytes, with Egger values of 0.010 and 0.036, respectively. The results of the combined effect of the number of oocytes and MII/mature oocytes did not change significantly using the trim and fill method (*P* = 0.767; *P* = 0.403), indicating that the non-significant result was relatively robust.

## 4. Discussion

Our systematic review and meta-analysis did not find effect of COVID-19 vaccines on biochemical pregnancy rates; number of oocytes and MII/mature oocytes obtained; implantation, blastocysts, and fertilization rates in women undergoing IVF treatment. Subgroup analysis showed that mRNA vaccine had no statistical significance on all indexes (clinical, biochemical, or ongoing pregnancy rates; implantation, blastocysts, or fertilization rates; and the number of oocytes and MII/mature oocytes). Notably, we found statistically significant differences in clinical and ongoing pregnancy rates between the vaccinated and unvaccinated groups. Through the elimination method (sensitivity test), Shi et al. (34) was found to be the main factor affecting the overall result (34). The age and body mass index (BMI) of the vaccinated group are higher than those of the unvaccinated group, and the number of people with pelvic fallopian tubes and ovulation disorders is larger than that of the unvaccinated group, suggesting that the physical conditions of pregnancy in the vaccinated group are worse than those in the unvaccinated group. Physical fitness is a very important factor affecting the process and outcome of IVF (39–41). Therefore, we speculated that this might be one of the reasons why the clinical pregnancy rate and ongoing pregnancy rate of the vaccinated group in the study of Shi et al. (34) was lower than those of the unvaccinated group. In addition, no statistical difference was observed in the subgroup analysis of the ongoing pregnancy rate between the vaccinated group and the unvaccinated group, but the overall difference was statistically significant, which may indicate that the effect of the vaccine on the ongoing pregnancy rate is uncertain, and more studies are needed to explore. The NOS quality of the included studies was relatively good in addition to a low risk of bias. We did not find publication bias in studies with clinical pregnancy rate analysis, and the publication bias in studies on the number of oocytes and MII/mature oocytes extracted did not change after the trim and fill method, indicating that the results of the study were stable.

Vaccination is the most effective preventative strategy against SARS-CoV-2 infection (42). However, misleading reports that COVID-19 vaccines may cause infertility or have an adverse effect on pregnancy have increased vaccination hesitancy in some women. Mi et al. (43) found that syncytin, a trapped retroviral envelope protein involved in human placental morphogenesis is primarily expressed in placental syncytial trophoblast cells (43). However, this does not suggest a possible homology between the vaccine-targeted SARS-CoV-2 spike protein and placental syncytin-1 that causes infertility (44). Administration of mRNA-1273 and BNT162b2 vaccines induces Th1 immunity in men and nonpregnant women, which elicits interferon-γ + CD8 + T-cell responses (45). However, the homeostasis of Th1/Th2 immunity regulates embryo implantation and pregnancy maintenance, thus raising concerns about the increased risk of pregnancy loss associated with COVID-19 vaccination (46). In addition, some misreports suggest that COVID-19 vaccines cause infertility in 97% of women and increases the risk of miscarriage, while negatively affecting both testicular and prostate testosterone levels (47). A large, phase III, multicenter, randomized controlled trial of mRNA-1273 vaccine found no safety concerns other than transient local and systemic reactions in subjects (48). In a multinational, randomized placebo-controlled trial evaluating the safety, efficacy, and immunogenicity of the BNT162b2 vaccine in adolescents and adults, adverse events were acceptable and thus, the vaccine was deemed safe. Despite multiple trials exploring COVID-19, almost all of these trials excluded pregnant women; however, vaccination during pregnancy can protect fetuses and newborn babies (49). In the V-safe Surveillance System and Pregnancy Registry, miscarriage (13.9%), preterm birth (9.4%), and small for gestational age (3.2%) were reported among participants who carried to term, but the rates were similar to those reported in pregnant populations studied before the COVID-19 pandemic (50). Studies of the safety and efficacy of COVID-19 vaccines suggest that they are safe, and the benefits would outweigh the risks of death and adverse pregnancy outcomes associated with SARS-CoV-2 infections (51).

Importantly, there are concerns about the effects of COVID-19 vaccination on pregnancy outcomes in women undergoing IVF. An initial analysis of these studies showed that vaccination against COVID-19 did not affect biochemical pregnancy rates; number of oocytes and MII/mature oocytes obtained; implantation, blastocysts, and fertilization rates after IVF. The studies we included mainly used two types of vaccines, inactivated vaccines and MRNA vaccines. Inactivated vaccines are produced using chemicals to inactivate viruses *in vitro*, keeping the viral particles intact as immunogens. mRNA vaccines are mRNA that is encapsulated by vector viral proteins or peptides (52, 53). Furthermore, subgroup analyses of the two main vaccines (mRNA and inactivated vaccines) administered to the study population. Subgroup analysis results showed that the mRNA vaccine does not affect the process (number of oocytes and MII/mature oocytes obtained; implantation, blastocysts, and fertilization rates) and outcome (clinical, biochemical and ongoing pregnancy rates) of IVF, but whether the inactivated vaccine affects the clinical pregnancy rate of IVF deserves more research to verify. Although the influence of inactivated vaccine on the clinical pregnancy rates is still unclear, considering that the COVID-19 vaccine can protect both mother and child, the probability of fetal infection with SARS-CoV-2 after birth can be reduced a certain extent (54, 55). Our analysis could help increase the willingness of women planning IVF treatment to receive COVID-19 vaccination, as well as provide evidence-based medical guidance for the development and implementation of guidelines. Age and BMI have an important impact on the course and outcome of IVF and should be accounted for when considering the results of our study. The number of oocytes and mature oocytes recovered from IVF is also related to age (56). A meta-analysis showed that female obesity had a significant negative impact on the live birth rate of IVF (57). Therefore, studies should pay attention to age and BMI matching between the experimental and control groups. Moreover, additional factors could affect the final pregnancy outcome after IVF, including differing IVF procedures in different countries and the expertise of different doctors should also be considered.

In this study, literature related to COVID-19 vaccines and IVF was thoroughly searched, and the studies that met the initial requirements were sorted through strict inclusion and exclusion criteria, and the heterogeneity of this meta-analysis was low. The quality of the included studies, which had a low risk of bias, was assessed using NOS. We also performed sensitivity analysis to verify the reliability of the results. The results with publication bias were meta-analyzed again using the trim-and-fill method, and the estimated pooled effect size did not change significantly, indicating that the results were relatively robust. The included studies were from Asia, Europe, and America; thus, the conclusions of our study are representative and universal.

Our study has several limitations. The number of oocytes and MII/mature oocytes in women undergoing IVF are related to individual ovarian reserves, thus, the effect of vaccination on oocyte number cannot be accurately determined. Moreover, the implantation and pregnancy outcomes are also affected by paternal factors, and pregnancy maintenance has external intervening factors. Therefore, a successful pregnancy is the result of interactions between several factors to provide a suitable environment, with numerous confounding factors. Some studies included in this meta-analysis were non-randomized retrospective studies because vaccination depended on patients' wishes, which made conducting prospective randomized clinical trial studies (RCTS) impossible. However, our meta-analysis included a large number of recent studies and provided robust results based on the random-effects model. Therefore, these results deserve attention.

## 5. Conclusion

Our findings suggest that vaccination against COVID-19 does not adversely affect the process (number of oocytes and MII/mature oocytes obtained; implantation, blastocysts, and fertilization rates) and outcome (biochemical pregnancy rates) of IVF. Subgroup analysis showed that the mRNA vaccine had no statistical significance on all indexes (clinical, biochemical, or ongoing pregnancy rates; implantation, blastocysts, or fertilization rates; and the number of oocytes and MII/mature oocytes). Whether inactivated vaccine affects clinical pregnancy rates need to be validated in high-quality prospective studies.

## Data availability statement

The original contributions presented in the study are included in the article/supplementary material, further inquiries can be directed to the corresponding author.

## Author contributions

Conceptualization: LZ, XS, RW, and FM. Data collection and analyses, writing – original draft preparation, and writing – original draft preparation: LZ, XS, and RW. Writing – review and editing: LZ, XS, and FM. LZ, XS, FM, and RW had primary responsibility for final content. All authors contributed to the article and approved the submitted version.
